# The Role of Anterior Insular Parvalbumin GABAergic Interneurons in Impulsivity‐Related and Alcohol‐Seeking Behaviors

**DOI:** 10.1002/cns.71138

**Published:** 2026-09-28

**Authors:** F. Bezerra, G. J. D. Fernandes, A. Anjos‐Santos, C. A. Favoretto, B. T. Rodolpho, P. Palombo, V. Vialou, K. P. Abrahao, F. C. Cruz

**Affiliations:** ^1^ Laboratory of Molecular and Behavioral Neuroscience, Departamento de Farmacologia Universidade Federal de São Paulo São Paulo Brazil; ^2^ Programa de Pós‐Graduação em Farmacologia, Escola Paulista de Medicina Universidade Federal de São Paulo São Paulo Brazil; ^3^ Sorbonne Université INSERM, CNRS, Neuroscience Paris Seine Paris France; ^4^ Departamento de Fisiologia, Escola Paulista de Medicina Universidade Federal de São Paulo São Paulo Brazil; ^5^ Programa de Pós‐Graduação em Psicobiologia, Escola Paulista de Medicina Universidade Federal de São Paulo São Paulo Brazil

**Keywords:** adolescence, alcohol, impulsivity, insular cortex, parvalbumin interneurons

## Abstract

**Introduction/Aim:**

Adolescence is characterized by heightened impulsivity and increased vulnerability to alcohol misuse. The insular cortex is critically involved in impulse control, and parvalbumin‐expressing (PV) GABAergic interneurons strongly regulate its activity. We investigated whether developmental differences in insular PV interneuron activity contribute to impulsivity‐related behaviors and alcohol consumption.

**Methods:**

Male C57BL/6 mice were assessed during adolescence (postnatal day 28) or young adulthood (postnatal day 64). Impulsivity was evaluated using the omission‐contingency learning (OCL) task, and alcohol intake and preference were measured using a two‐bottle choice paradigm with quinine adulteration. Neuronal activation in the insular cortex was analyzed using Fos immunolabeling combined with NeuN or PV markers. Chemogenetic manipulation of insular PV interneurons was performed in PV‐Cre mice.

**Results:**

Adolescent mice exhibited greater impulsivity and higher alcohol consumption than adults. Although overall neuronal activation in the insular cortex was increased in adolescents, PV interneuron activation was reduced. Chemogenetic activation of insular PV interneurons decreased impulsive responding and alcohol intake, whereas inhibition produced the opposite effects.

**Conclusion:**

Insular PV interneurons exert critical control over impulsivity and alcohol‐seeking behaviors. Developmental differences in PV‐mediated inhibitory regulation within the insular cortex may underlie adolescent vulnerability to alcohol misuse.

Adolescent impulsivity is linked to increased alcohol seeking and altered activation of anterior insular PV interneurons. Chemogenetic modulation of these neurons regulates inhibitory control and alcohol‐related behaviors, highlighting their role in vulnerability to alcohol use disorder.

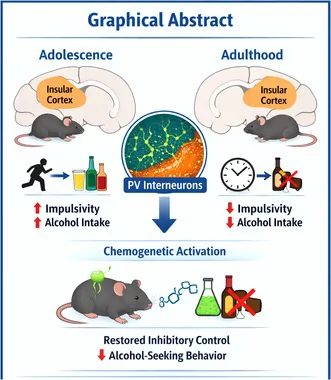

## 1 Introduction

Alcohol use remains one of the most widespread forms of psychoactive substance misuse worldwide and continues to represent a major public health challenge [1, 2, 3]. Alcohol use disorder (AUD) is characterized by compulsive intake, impaired behavioral control, and persistent consumption despite adverse consequences [4, 5, 6]. Importantly, alcohol use often begins during adolescence, a developmental period marked by substantial neurobiological remodeling and heightened vulnerability to maladaptive behaviors [7]. Adolescence is associated with increased risk‐taking, emotional reactivity, and impulsivity, reflected by a preference for immediate rewards and reduced inhibitory control [8, 9, 10]. Exposure to alcohol during this critical window may exacerbate these vulnerabilities, reinforcing reward‐driven behaviors and increasing the risk for the development of AUD [11, 12]. Consistent with this, impulsivity has been strongly associated with the initiation and escalation of alcohol use, with more impulsive adolescents showing a greater likelihood of hazardous drinking later in life [13, 14, 15, 16].

At the neural level, the insular cortex acts as an integrative hub for interoceptive, affective, and cognitive processing, playing a central role in decision‐making and behavioral control. Its extensive connectivity with cortical and subcortical regions supports its involvement in impulsivity‐related behaviors [17, 18, 19]. Clinical and preclinical evidence indicate that functional alterations in the insular cortex are associated with impulsivity and impaired behavioral regulation [20, 21]. During adolescence, ongoing structural and functional maturation of the insula has been linked to individual differences in impulsivity, with reduced neuronal excitability in the anterior insula associated with increased impulsive and compulsive behaviors [22, 23, 24]. In contrast, experimental activation of this region has been shown to attenuate such behaviors [25].

The insular cortex has emerged as a critical cortical hub in the regulation of alcohol‐related behaviors, integrating interoceptive, emotional, and motivational signals that influence both alcohol consumption and relapse vulnerability [26]. Growing evidence indicates that the anterior insular cortex, in particular, plays a central role in compulsive alcohol seeking, as pharmacological or chemogenetic manipulations of this region significantly alter the propensity to relapse following punishment‐imposed abstinence, highlighting its importance in alcohol seeking, despite adverse consequences [27]. In parallel, structural adaptations within insular microcircuits have also been implicated in compulsive drinking, as repeated binge‐like ethanol exposure increases perineuronal net expression in the insular cortex, and disruption of these extracellular matrix structures reduces aversion‐resistant alcohol drinking, directly linking insular plasticity to compulsive ethanol consumption [28, 29]. Recent evidence highlights that functional heterogeneity across insular subregions critically contributes to compulsive drinking, withdrawal‐related negative affect, and relapse‐like behaviors, positioning the anterior insular cortex as a key neural substrate underlying maladaptive alcohol‐seeking behaviors [26].

Within the anterior insular cortex, behavioral regulation depends on a precise balance between excitatory pyramidal neurons and inhibitory GABAergic interneurons [30, 31]. Parvalbumin‐positive (PV) interneurons provide fast and powerful perisomatic inhibition, shaping pyramidal neuron output and supporting coordinated cortical activity [32]. Disruptions in excitation–inhibition balance are implicated in multiple neuropsychiatric conditions characterized by impaired impulse control [33, 34]. Chemogenetic studies have demonstrated that PV interneurons in prefrontal and cingulate regions modulate reward‐seeking behavior, compulsivity, and impulsivity [35, 36]. In the insula, alterations in PV interneuron density or myelination have been associated with impaired danger recognition and enhanced pursuit of immediate rewards [34, 37]. However, the contribution of insular PV interneurons to impulsivity during adolescence remains poorly understood.

Therefore, the present study aimed to investigate the role of PV interneurons in the insular cortex during adolescence, a critical developmental stage characterized by heightened impulsivity and increased vulnerability to alcohol use disorder.

## 2 Materials and Methods

### 2.1 Animals

All experiments were conducted using male mice on a C57BL/6J background. Experiments 1 and 2 were performed in wild‐type C57BL/6J mice, whereas Experiments 3 and 4 were conducted in PV‐Cre transgenic mice. PV‐Cre mice (B6;129P2‐Pvalbtm1(cre)Arbr/J), as previously described [38], were obtained from the Centro de Desenvolvimento de Modelos Animais para a Biologia e Medicina (CEDEME, Universidade Federal de São Paulo) or The Jackson Laboratory and backcrossed onto a C57BL/6J background.

Animals were tested during adolescence (postnatal days 28–47) or young adulthood (postnatal day 64) [39]. Mice were housed under a reverse 12‐h light/dark cycle in a temperature‐ and humidity‐controlled facility and were maintained at approximately 85% of their baseline body weight during behavioral testing. All experimental procedures were approved by the Comissão de Ética no Uso de Animais at Universidade Federal de São Paulo (CEUA #9191091219) and were conducted in accordance with the European Directive 2010/63/EU.

### 2.2 Omission‐Contingency Learning (OCL) Test

The omission‐contingency learning (OCL) task was conducted in operant conditioning chambers (Med Associates) equipped with an active nose‐poke aperture, a cue light, a house light, and a reward magazine. Animals were initially trained to establish a stable operant response under progressively increasing fixed‐ratio reinforcement schedules, with three consecutive training sessions conducted at each schedule (FR1, FR3, and FR5). This was followed by a 30‐min OCL test session. During the training phase, responses were reinforced according to fixed‐ratio schedules, with no omission contingency. During the test session, sucrose pellets were delivered according to a fixed 30‐s interval, but any response emitted during this interval reset the timer and delayed reward delivery. Thus, the contingency shifted from action‐dependent reinforcement during training to omission‐based reinforcement during testing. This omission‐contingency procedure assesses inhibitory control and behavioral flexibility by requiring animals to suppress a previously reinforced response and adapt to a changed action–outcome relationship. The primary behavioral measures were the total number of active nose‐pokes (ANP) and rewards earned (REW), with interval‐resetting responses additionally quantified as a direct measure of inappropriate responding.

### 2.3 Two‐Bottle Choice and Quinine‐Adulterated Drinking Sessions

Alcohol consumption and preference were assessed using a two‐bottle choice paradigm. Mice were given concurrent access to water and a 10% (v/v) ethanol solution during daily 4‐h sessions. After six baseline sessions, ethanol was adulterated with increasing concentrations of quinine (0.6 mM–1.0 mM) across three sessions, followed by three additional ethanol‐only sessions. Alcohol intake (g/kg) and preference were calculated for each session.

### 2.4 Immunofluorescence and Immunohistochemistry

Neuronal activation in the insular cortex was assessed using Fos immunoreactivity combined with NeuN or parvalbumin (PV) labeling. Immunofluorescence was used to quantify the percentage of activated neurons and PV interneurons, whereas immunohistochemistry was used to quantify Fos‐positive nuclei. Image acquisition and quantification were performed in a blinded manner using standardized imaging parameters.

### 2.5 Surgical Procedures

Stereotaxic surgeries were performed in PV‐Cre mice at postnatal day 35 under isoflurane anesthesia. Cre‐dependent AAV vectors encoding hM3D(Gq)‐mCherry, hM4D(Gi)‐mCherry, or eYFP were injected unilaterally into the anterior insular cortex. Viral expression was verified histologically, and injections were counterbalanced between hemispheres to control for lateralization effects [40, 41]. Chemogenetic manipulation was achieved via systemic administration of clozapine N‐oxide (CNO).

Detailed protocols, information, and image processing procedures are provided in the Supplementary Methods.

### 2.6 Experimental Design

Experiment 1 assessed impulsivity and alcohol‐related behaviors in adolescent and adult mice using the omission‐contingency learning (OCL) task followed by two‐bottle choice drinking sessions (Figure 1A). A total of 59 animals were used, including 29 adolescents and 30 adults.

**FIGURE 1:**
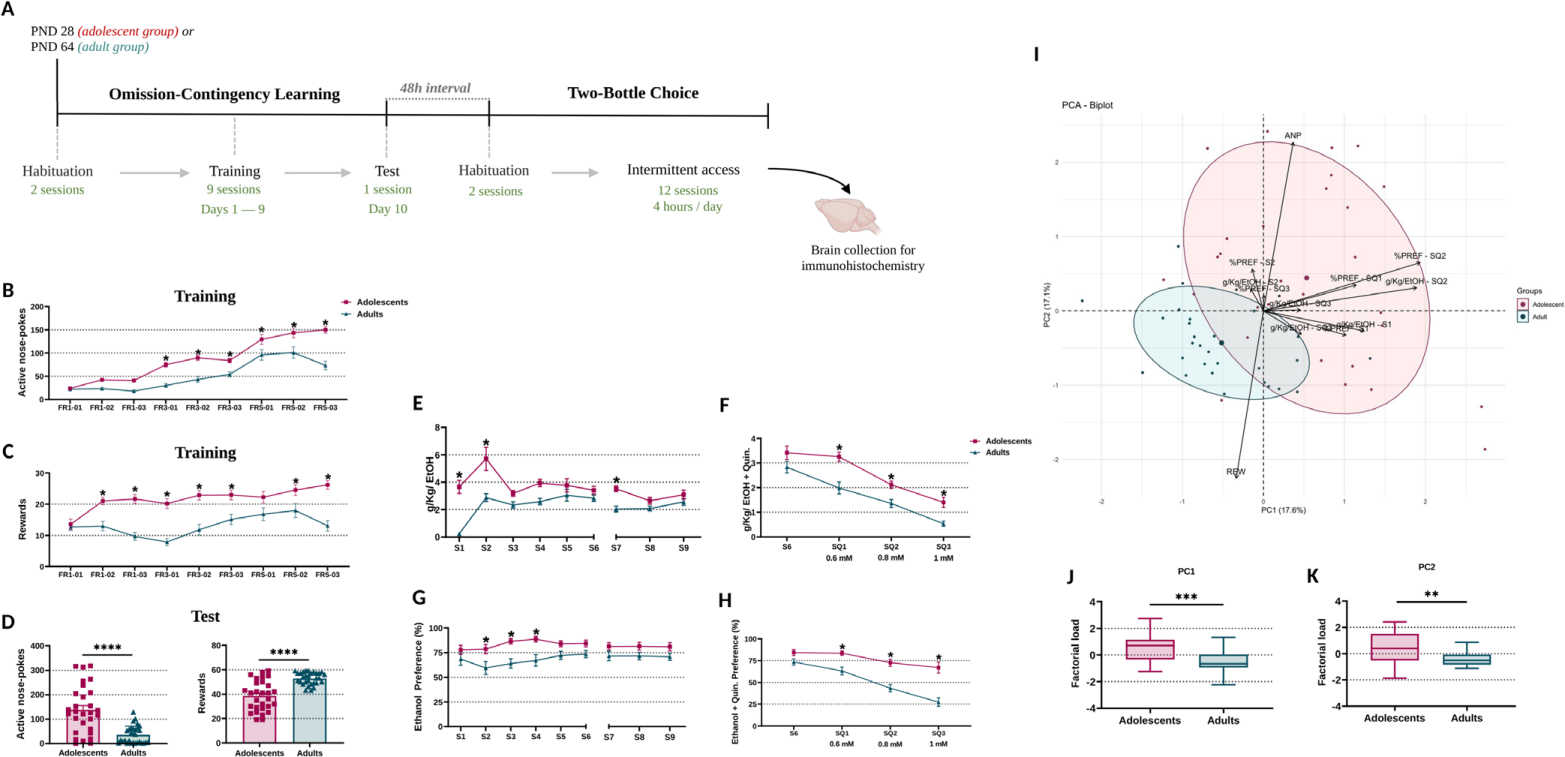
Omission‐contingency learning and ethanol preference in adolescent and adult mice. (A) Schematic representation of the experimental design. Adolescent and adult mice underwent omission‐contingency learning (OCL) training across nine consecutive sessions (Days 1–9), followed by behavioral testing on Day 10. Animals were subsequently evaluated in the two‐bottle choice paradigm, including ethanol consumption, quinine‐adulterated ethanol consumption, and ethanol preference assessments, as described in the Methods section. (B, C) Training phase of the OCL task. Adolescent mice displayed increased active nose‐pokes (ANP) and rewards earned compared with adults across training sessions. (D) Test phase of the OCL task showing increased ANP and reduced rewards earned in adolescent mice, indicating impaired inhibitory control during omission‐contingency testing. (E, F) Ethanol intake (g/kg) during baseline ethanol sessions (S1–S6 and S7–S9) and during quinine‐adulterated ethanol sessions (SQ1–SQ3). Quinine concentrations are indicated on the x‐axis. (G, H) Ethanol preference (%) during baseline ethanol sessions and quinine‐adulterated ethanol preference (%) during SQ1–SQ3 sessions. (I) Principal component analysis (PCA) showing segregation of behavioral variables between adolescent and adult groups. (J, K) Variable loadings for PC1 and PC2, demonstrating stronger associations between ANP and rewards earned in adolescent mice compared with adults. Data are presented as mean ± SEM. Adolescents: *N* = 29; adults: *N* = 30. Statistical analyses were performed using two‐way ANOVA or one‐way ANOVA followed by appropriate post hoc tests. *p < 0.05, **p < 0.01, ***p < 0.001.

Experiment 2 examined insular cortical activation following the OCL task using Fos‐based immunolabeling (Figure 2A), using a separate cohort of animals (*n* = 15 per group).

**FIGURE 2:**
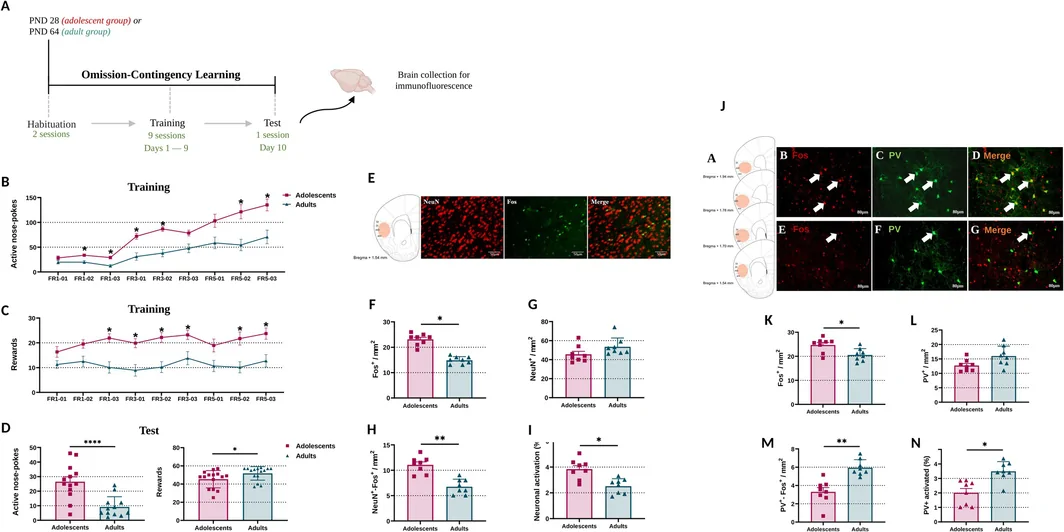
Insular cortex neuronal and parvalbumin interneuron activation following omission‐contingency learning. (A) Experimental design for omission‐contingency learning (OCL) followed by immunohistochemical and immunofluorescence analyses of the insular cortex. Adolescent and adult mice were trained and tested in the OCL task, and brain tissue was collected 1 h after testing. (B, C) During OCL training, adolescent mice exhibited increased active nose‐pokes (ANP) and rewards earned compared with adults across sessions. (D) During the OCL test session, adolescent mice displayed increased ANP and reduced rewards earned relative to adults. (E) Representative confocal images of insular cortex sections showing NeuN‐positive neurons (red), Fos‐positive cells (green), and merged Fos+/NeuN+ co‐localization (yellow). Scale bar = 80 μm. (F–I) Quantification of neuronal activation in the insular cortex. (F) Number of Fos‐positive cells. (G) Number of NeuN‐positive cells. (H) Number of Fos+/NeuN+ double‐labeled cells. (I) Percentage of activated neurons (Fos+/NeuN+ relative to total NeuN+ cells). (J) Atlas representation of the analyzed insular cortex subregions (orange). Representative immunofluorescence images from adolescent and adult mice showing DAPI staining (blue), parvalbumin‐positive (PV+) interneurons (green), Fos‐positive cells (red), and merged Fos+/PV+ co‐localization. Arrows indicate double‐labeled cells. Scale bar = 80 μm. (K–N) Quantification of PV interneuron activation in the insular cortex. (K) Number of Fos‐positive cells. (L) Number of PV+ cells. (M) Number of Fos+/PV+ double‐labeled cells. (N) Percentage of activated PV interneurons. Data are presented as mean ± SEM. Behavioral analyses: *N* = 15 per group. Immunohistochemical analyses: *N* = 8 per group. Statistical analyses were performed using two‐way ANOVA or one‐way ANOVA followed by appropriate post hoc tests. *p < 0.05, **p < 0.01, ***p < 0.005, ****p < 0.0001.

Experiment 3 evaluated the causal role of insular PV interneurons in impulsivity using chemogenetic manipulation in PV‐Cre mice, followed by behavioral testing and immunofluorescence analysis (Figure 3A), with animals assigned to Gq, Gi, and control groups (*n* = 13 per group).

**FIGURE 3:**
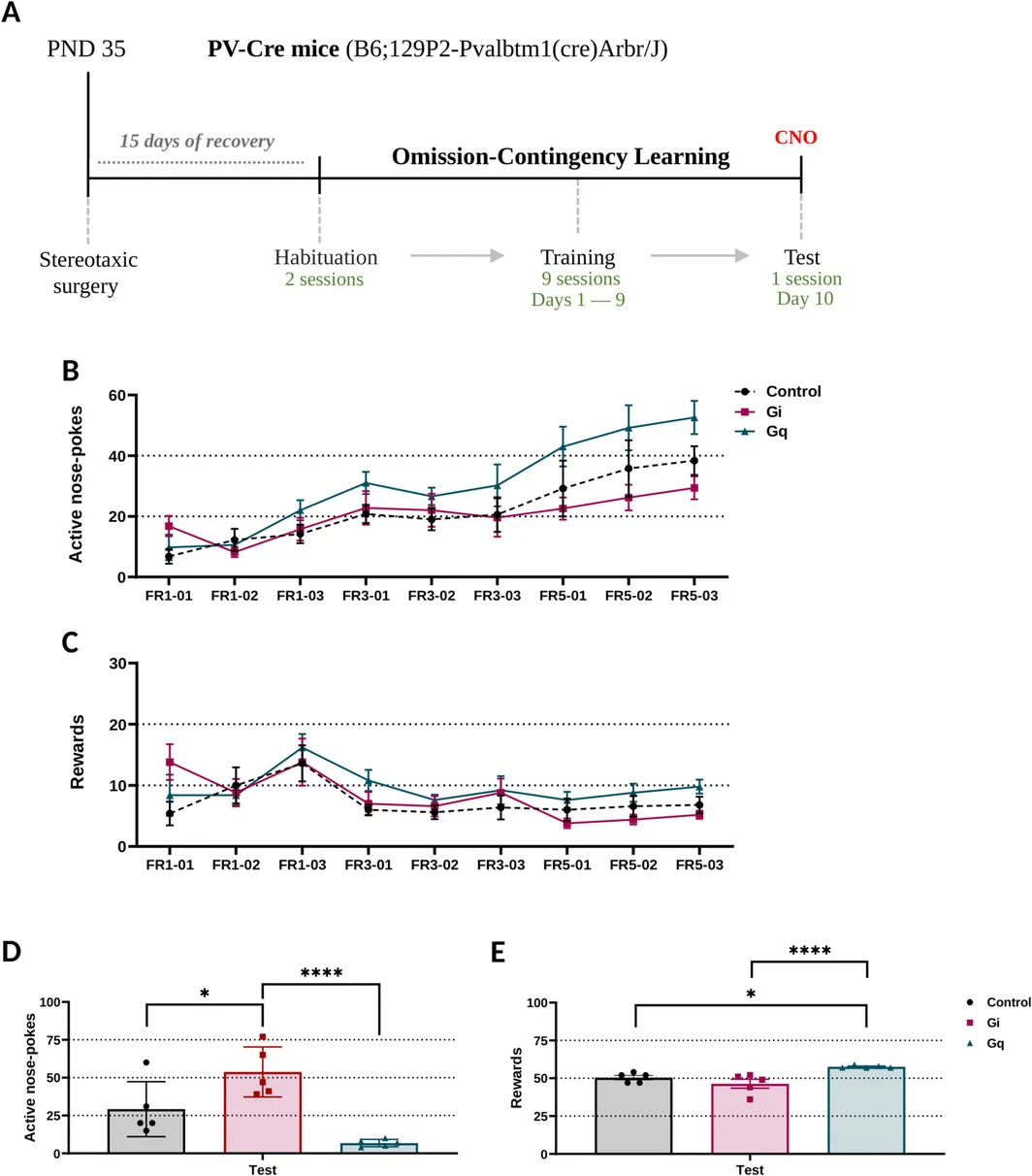
Chemogenetic modulation of insular cortex PV interneurons during omission‐contingency learning. (A) Schematic representation of the experimental design for chemogenetic manipulation during omission‐contingency learning. PV‐Cre mice (B6;129P2‐Pvalbtm1(cre)Arbr/J) received stereotaxic injections of Cre‐dependent DREADD vectors targeting the anterior insular cortex, followed by a recovery period and behavioral testing under clozapine‐N‐oxide (CNO) administration. The timeline indicates training sessions across Days 1–9 and behavioral testing on Day 10. (B, C) Training phase of the OCL task showing active nose‐pokes (ANP) and rewards earned across sessions in control, inhibitory DREADD (Gi), and excitatory DREADD (Gq) groups. (D, E) Test phase of the OCL task. Chemogenetic inhibition of PV interneurons increased ANP and reduced rewards earned, whereas chemogenetic activation produced the opposite behavioral profile. Data are presented as mean ± SEM. Control DREADD: Black; Gi DREADD: Red; Gq DREADD: Blue. *n* = 5 per group. Statistical analyses were performed using two‐way ANOVA or one‐way ANOVA followed by appropriate post hoc tests. *p < 0.05, **p < 0.01, ****p < 0.0001.

Experiment 4 examined ethanol‐related behaviors following chemogenetic manipulation of insular PV interneurons (Figure 4A), using *n* = 8 animals per group.

**FIGURE 4:**
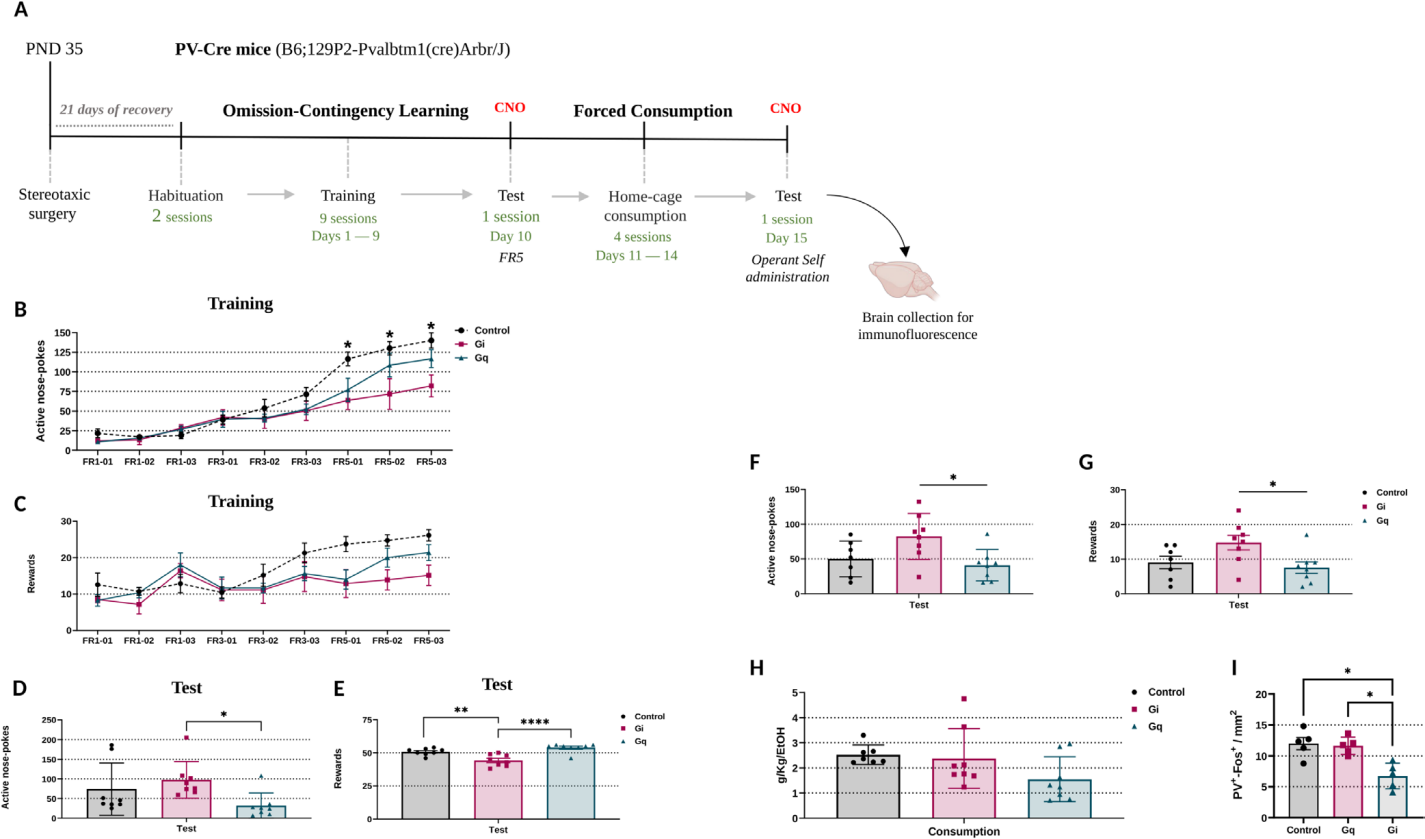
Chemogenetic modulation of insular cortex PV interneurons alters ethanol self‐administration and neuronal activation. (A) Experimental design for chemogenetic manipulation during omission‐contingency learning combined with ethanol intake procedures. PV‐Cre mice received stereotaxic injections of Cre‐dependent DREADD vectors into the anterior insular cortex, followed by recovery, CNO administration, operant training, forced ethanol consumption, and ethanol self‐administration testing, as detailed in the Methods section. (B, C) During OCL training, mice expressing excitatory DREADDs (Gq) displayed increased active nose‐pokes (ANP) and rewards earned compared with control and inhibitory DREADD (Gi) groups. (D, E) During the OCL test phase, chemogenetic inhibition of PV interneurons reduced ANP and rewards earned, whereas activation of PV interneurons increased behavioral responding. (F, G) Operant ethanol self‐administration. Chemogenetic inhibition of PV interneurons reduced ethanol‐seeking behavior and rewards earned compared with Gq mice. (H) Ethanol intake during the final habituation session showed no significant differences between groups. (I) Quantification of Fos+/PV+ double‐labeled cells in the insular cortex following ethanol self‐administration. Chemogenetic inhibition reduced PV interneuron activation, whereas chemogenetic activation increased Fos+/PV+ co‐localization. Data are presented as mean ± SEM. Behavioral analyses: *N* = 8 per group. Immunohistochemical analyses: *N* = 5 per group. Statistical analyses were performed using two‐way ANOVA or one‐way ANOVA followed by appropriate post hoc tests. *p < 0.05, **p < 0.01, ***p < 0.001, ****p < 0.0001.

Detailed protocols, antibody information, and image processing procedures are provided in the Supplementary Methods.

### 2.7 Data Analysis

All datasets were first assessed for normality using the D'Agostino–Pearson omnibus test and for homogeneity of variances using the F‐test. Non‐parametric comparisons were performed using the Mann–Whitney U test. For normally distributed datasets with multiple factors or repeated measures, a one‐way or two‐way repeated‐measures ANOVA followed by Bonferroni *post hoc* tests was used. Animals were excluded (*n* = 7) only when identified as statistical outliers based on Grubbs' test. All results are presented as mean ± standard error of the mean (SEM), and statistical significance was set at *p* < 0.05, *p* < 0.01, or *p* < 0.001.

For Experiment 1, training performance across sessions was analyzed using a two‐way ANOVA (ontogenic period × session) with Bonferroni *post hoc* adjustments. OCL test outcomes were evaluated using the Mann–Whitney U test. Before performing the principal component analysis (PCA), a Pearson correlation matrix was constructed from all OCL and two‐bottle choice variables. Variables exhibiting high correlations (|*r*| > 0.8) were excluded to reduce redundancy. The PCA was then conducted using the remaining variables, and components were retained based on eigenvalues > 1.

For Experiment 2, the training data were analyzed using a two‐way ANOVA (ontogenic period × session), followed by Bonferroni *post hoc* tests. OCL test measures and neuronal activation data were analyzed using the Mann–Whitney U test.

For Experiment 3, OCL training data were analyzed using a two‐way ANOVA (DREADD group × session), followed by Bonferroni *post hoc* comparisons. OCL test outcomes, ethanol‐seeking behavior, and locomotor activity were analyzed using one‐way ANOVA followed by Tukey's *post hoc* test.

All statistical analyses were performed using SPSS (version 25.0.0) and Jamovi (version 1.2.27). Graphs were generated using GraphPad Prism 8 (version 8.0.2). The significance threshold for all analyses was α = 0.05.

## 3 Results

### 3.1 Experiment 1: Adolescent Mice Exhibit Greater Impulsivity‐Related Behaviors and Ethanol Consumption Than Adults

To determine whether adolescent mice exhibit heightened impulsivity and increased ethanol consumption compared to adults, we first analyzed their performance during Omission‐Contingency Learning (OCL) training sessions. For active nose‐pokes (ANP; Figure 1B), two‐way ANOVA revealed a significant main effect of session [F(8,456) = 102; *p* < 0.0001], a significant main effect of ontogenic period [F(1,57) = 28.16; *p* < 0.0001], and a significant ontogenic period × session interaction [F(8,456) = 7.678; *p* < 0.0001]. *Post hoc* comparisons revealed that adolescents made significantly more ANPs than adults across all FR3 sessions (*p* < 0.05) and all FR5 sessions (*p* < 0.01), whereas no differences were observed during FR1 training (*p* > 0.05), indicating impaired inhibitory control under higher response demands (adolescents > adults during FR3 and FR5; adolescents = adults during FR1).

For the number of rewards (Figure 1C), two‐way ANOVA likewise revealed a significant main effect of session [F(8,456) = 11.94; *p* < 0.0001], a significant main effect of ontogenic period [F(1,57) = 25.48; *p* < 0.0001], and a significant ontogenic period × session interaction [F(8,456) = 6.322; *p* < 0.0001]. Post hoc analysis demonstrated that adolescents earned more rewards than adults during FR3 and FR5 training sessions (*p* < 0.05), except in the first FR3 session, in which reward acquisition did not differ between age groups (adolescents > adults during FR3 and FR5; adolescents = adults during the first FR3 session). In the OCL test session, Mann–Whitney analyses demonstrated robust age‐related differences in both ANP and REW measures (*p* < 0.0001 for both), indicating impaired operant inhibition in adolescents compared to adults (adolescents > adults during FR3 and FR5; adolescents = adults during FR1) (Figure 1D).

Notably, while adolescent mice showed intact acquisition of the operant response during training, their impaired performance during the OCL test suggests a deficit in updating action–outcome contingencies following the shift from action–reward to omission–reward. This pattern is consistent with reduced behavioral flexibility rather than impaired learning per se.

During the two‐bottle choice procedure, adolescents consumed significantly more ethanol than adults. For total ethanol consumption (g/Kg of EtOH), the ANOVA revealed significant differences for the session factor [F(8,456) = 7.413; *p* < 0.0001], the ontogenic period factor [F(1,57) = 78.77; *p* < 0.001], and a significant interaction between factors (ontogenic period × session) [F(8,456) = 4.707; *p* < 0.001], with post hoc differences observed in sessions S1, S2, and S7 (*p* < 0.0001; Figure 1E). These results indicate that ethanol intake was higher in adolescents than adults during early drinking sessions and following the quinine phase (adolescents > adults). During quinine‐adulterated ethanol access (g/Kg of EtOH), the analysis revealed a significant difference for the session factor [F(3,171) = 48.01; *p* < 0.0001], the ontogenic period factor [F(1,57) = 29.83; *p* < 0.0001], with no significant interaction between the factors [F(3,171) = 1.088; *p* > 0.05], adolescents consumed more ethanol across all quinine sessions (Figure 1F). These findings suggest that ethanol intake persists longer in adolescent mice despite aversive adulteration (adolescents > adults). Analyses of ethanol preference similarly revealed heightened ethanol motivation in adolescents. For ethanol preference in EtOH periods 1 (S1–S6) and 2 (S7–S9), ANOVA revealed no significant difference for the session factor [F(8,456) = 0.9662; *p* = 0.4618], a significant difference for the ontogenic period factor [F(1,57) = 33.82; *p* < 0.0001], with no significant interaction between the factors [F(8,456) = 0.8097; *p* = 0.5943] (Figure 1G). During the quinine phase (SQ1–SQ3), the analysis again revealed a significant main effect of the session factor [F(3,171) = 23.60; *p* < 0.0001], a significant main effect of ontogenic period factor [F(1,57) = 56.93; *p* < 0.0001], and a significant interaction between factors (ontogenic period × session) [F(3,171) = 4.255; *p* = 0.0063], with post hoc differences observed across all quinine sessions (*p* < 0.001; Figure 1H). These findings indicate that adolescents displayed greater ethanol preference than adults across all drinking conditions (adolescents > adults).

To identify latent behavioral dimensions underlying age‐dependent differences in ethanol‐related behaviors, we performed a principal component analysis (PCA) using variables derived from the omission‐contingency learning (OCL) task and the two‐bottle choice procedure (Figure 1I). Twelve components were initially extracted, and components with eigenvalues greater than 1 were retained according to the Kaiser criterion. This yielded five principal components, which together explained 73.1% of the total variance, indicating that a substantial proportion of the behavioral variability was captured by a limited number of underlying dimensions. The first principal component (PC1; 17.6% of variance explained, eigenvalue = 3.13) showed strong positive loadings for variables related to early ethanol consumption and quinine‐adulterated ethanol intake (e.g., S1 and SQ2), suggesting that this component reflects an “early compulsive drinking” dimension, characterized by elevated ethanol consumption despite aversive adulteration. PC1 scores were significantly higher in adolescent mice compared with adults (*p* < 0.0001; Figure 1J), indicating greater engagement of this behavioral domain during adolescence. The second principal component (PC2; 17.1%, eigenvalue = 1.66) was primarily loaded by OCL‐related measures, including active responses and reinforcement‐associated variables, defining a behavioral dimension associated with inhibitory control and action suppression under contingency change. PC2 also significantly differed between age groups (*p* < 0.05; Figure 1K), consistent with age‐dependent differences in behavioral flexibility and impulse control. The third and fourth components (PC3, 12.7%; PC4, 12.0%) were mainly associated with ethanol intake during later quinine‐adulteration sessions, likely reflecting sustained aversion‐resistant drinking. However, these components did not significantly differ between adolescents and adults, suggesting comparable maintenance of this behavioral phenotype across age groups. Finally, the fifth principal component (PC5; 8.5%, eigenvalue = 1.03) was predominantly associated with early two‐bottle choice sessions, representing an “initial ethanol preference” dimension. PC5 scores were significantly elevated in adolescent mice compared with adults (*p* < 0.001; Figure S1). Collectively, these analyses reveal that adolescence is characterized by stronger clustering of behavioral traits related to impaired inhibitory control, elevated ethanol consumption, and aversion‐resistant drinking, supporting the existence of an age‐dependent behavioral phenotype associated with vulnerability to compulsive alcohol use.

Across components, adolescents showed stronger covariance among impulsivity measures and ethanol intake, even under aversive conditions.

Finally, the Mann–Whitney test performed on Fos immunohistochemistry data obtained after the final ethanol session (S9) revealed no age‐related differences in total Fos‐positive neuronal counts in the insular cortex (*p* > 0.5) (Figure S2, S3).

### 3.2 Experiment 2: Impaired Inhibitory Control in Adolescent Mice Is Associated With Altered Activation of the Insular Cortex

To investigate the neural correlates underlying age‐dependent impulsivity and ethanol‐related behaviors, we quantified neuronal activation in the anterior insular cortex following completion of the OCL task (Figure 2B–D). Fos immunoreactivity was assessed to evaluate overall neuronal activation (NeuN+/Fos+) (Figure 2E) as well as activation within parvalbumin‐positive (PV+) GABAergic interneurons (Figure 2A). Adolescent mice exhibited significantly greater Fos expression in the insular cortex compared with adults (*p* < 0.001; Figure 2F), indicating increased global neuronal activation (adolescents > adults). Consistent with this, the number of double‐labeled NeuN+/Fos + neurons was also higher in adolescents (*p* < 0.01, Figure 2H), resulting in a greater percentage of activated neurons overall (*p* < 0.05; adolescents > adults; Figure 2I).

To evaluate PV interneuron activation, we quantified the proportion of PV+ cells co‐expressing Fos (PV+/Fos+) (Figure 2J). Only a small fraction of PV interneurons was activated in both age groups (Figure 2M). Adolescents showed a lower percentage of activated PV interneurons (1.87 ± 0.14) relative to adults (2.92 ± 0.62), despite exhibiting a higher total number of Fos‐labeled cells overall (*p* < 0.05; adults > adolescents). The total number of PV+ interneurons did not differ between groups (*p* > 0.05; Figure 2L). In contrast, adults displayed a significantly greater number of PV+/Fos + neurons (*p* < 0.01) (Figure 2K) and a higher percentage of PV interneuron activation (*p* < 0.05; Figure 2N) compared with adolescents (adults > adolescents).

Together, these findings suggest that, while adolescents exhibit increased global neuronal activation in the insular cortex, adults display preferential engagement of PV interneurons. This divergence suggests developmental differences in inhibitory circuit recruitment that may contribute to heightened impulsivity and ethanol‐related behaviors during adolescence.

### 3.3 Experiment 3.1: Activation of Parvalbumin‐Positive Interneurons in the Insular Cortex Enhances Inhibitory Control

To directly assess the causal contribution of PV+ GABAergic interneurons in the insular cortex to impulsivity‐related behaviors, we expressed Cre‐dependent DREADDs (Gq‐ or Gi‐coupled human muscarinic receptors) in PV‐Cre transgenic mice. We evaluated their performance in the OCL task. Thirty minutes before testing, mice received an intraperitoneal injection of CNO (1 mg/kg), a dose shown to activate both Gq‐ and Gi‐DREADDs effectively.

During OCL training, two‐way ANOVA on active nose‐pokes (ANP) indicated a significant difference for the sessions factor [F(8,96) = 20.99; *p* < 0.0001], no significant difference for the DREADD factor [F(2,12) = 2.711; *p* = 0.1068], and a significant interaction between factors [F(16,96) = 1.833; *p* = 0.0374]. Post hoc analysis indicated no differences between groups across sessions (*p* > 0.05; Figure 3B) (Gq = Gi = Control during training). Similarly, two‐way ANOVA for the number of rewards indicated a significant difference for the sessions factor [F(8,96) = 6.191; *p* < 0.0001], no significant difference for the DREADD factor [F(2,12) = 1.120; *p* = 0.3581], and no significant interaction between factors [F(16,96) = 1.245; *p* = 0.2494]. Post hoc analysis indicated no differences between groups across sessions for the number of rewards (*p* > 0.05; Figure 3C) (Gq = Gi = Control during training).

In the OCL test session, however, chemogenetic manipulation produced robust behavioral effects. For ANP, two‐way ANOVA revealed a significant difference for the session factor [F(1,12) = 13.11; *p* = 0.0035], no significant difference for the DREADD factor [F(2,12) = 1.434; *p* = 0.2764], and a significant interaction between factors [F(2,12) = 51.77; *p* < 0.0001]. Post hoc comparisons showed that Gi‐DREADD inhibition of PV interneurons increased impulsive responding (higher ANP) relative to Control (*p* < 0.05) and Gq groups (*p* < 0.001), whereas Gq‐DREADD activation was associated with reduced impulsive responses (Figure 3D). For REW, two‐way ANOVA revealed a significant difference for the session factor [F(1,12) = 860.2; *p* < 0.0001], a significant difference for the DREADD factor [F(2,12) = 22.49; *p* < 0.0001], and no significant interaction between factors [F(2,12) = 1.638; *p* = 0.2350]. Reward outcomes were directionally consistent with ANP findings, with Gq activation decreasing reinforcement acquisition relative to both Control and Gi groups (Figure 3E) (Gi > Control > Gq for impulsivity).

These findings demonstrate that the activity of insular PV+ interneurons exerts bidirectional control over impulsive responding. Enhancing PV interneuron activity (Gq‐DREADDs) is associated with improved inhibitory control by suppressing inappropriate nose‐pokes, whereas inhibiting these interneurons (Gi‐DREADDs) is associated with impaired inhibitory control and increased impulsive behavior. Overall, PV interneurons in the insular cortex play a critical modulatory role in regulating impulse‐related actions during the OCL task.

### 3.4 Experiment 4: Chemogenetic Modulation of PV+ Interneurons in the Insular Cortex Alters Ethanol‐Seeking Responses

To determine whether insular PV+ GABAergic interneurons regulate ethanol‐seeking behavior, PV‐Cre mice injected with AAV‐EF1a‐DIO‐hM3Dq or control vectors (*n* = 8 per group) were subjected to a forced‐ethanol consumption protocol (12 h/6 h/4 h/2 h). Behavioral testing was conducted 30 min following CNO administration (1 mg/kg; Figure 4A) (Gq = Gi = Control).

During OCL training, two‐way ANOVA on active nose‐pokes (ANP) indicated a significant difference for the sessions factor [F(8,96) = 20.99; *p* < 0.0001], no significant difference for the DREADD factor [F(2,12) = 2.711; *p* = 0.1068], and a significant interaction between factors [F(16,96) = 1.833; *p* = 0.0374]. Post hoc analysis indicated differences between the Control and Gq groups for the FR5‐01, 02 and 03 sessions (*p* < 0.05) (Figure 4B). For rewards (REW), ANOVA revealed a significant difference for the sessions factor [F(8,96) = 6.191; *p* < 0.0001], no significant difference for the DREADD factor [F(2,12) = 1.120; *p* = 0.3581], and no significant interaction between the factors [F(16,96) = 1.245; *p* = 0.2494]. However, the post hoc analysis did not demonstrate differences between the groups throughout the sessions for the number of rewards (*p* > 0.05) (Figure 4C).

In the OCL test, ANOVA indicated significant group effects for ANP [F(2,21) = 5.852; *p* = 0.0146], with post hoc analysis showing increased impulsive response in Gi relative to Gq mice (*p* < 0.05; Figure 4D). For REW, ANOVA also revealed significant group differences [F(2,21) = 15.59; *p* < 0.0001], with Control vs. Gi (*p* < 0.01) and Gi vs. Gq (*p* < 0.0001) pairs differing significantly (Figure 4E). During the final forced‐ethanol exposure session (T = 2 h), ethanol consumption (g/kg EtOH) did not differ between groups [F(2,9) = 2.401; *p* = 0.146] (Figure 4H). (Gq = Gi = Control). Mice were then tested in an operant ethanol self‐administration session following prior operant training and ethanol exposure. This session was used as a behavioral probe of alcohol‐directed responding rather than a model of acquisition or maintenance of self‐administration.

During the ethanol self‐administration test, ANOVA revealed significant group differences for both ANP [F(2,20) = 4.905; *p* < 0.05] and REW [F(2,20) = 4.286; *p* = 0.0282]. Post hoc analysis revealed significant differences between Gi and Gq for both outcomes (*p* ≈0.02–0.03; Figure 4F,G) (Gi>Gq for ethanol seeking).

To rule out nonspecific effects of CNO on locomotor activity, we performed an open‐field assessment. No significant group differences were observed in total distance traveled [F(2,9) = 0.7814; *p* = 0.4865] or average speed [F(2,9) = 2.401; *p* = 0.1460] (Figure S4).

Finally, to evaluate PV interneuron activation, we quantified the proportion of PV+ cells co‐expressing Fos (PV+/Fos+). ANOVA revealed significant group differences [F(2,12) = 11.47; *p* < 0.01], with *post hoc* comparisons showing significant differences between the Gi vs. Gq groups (*p* < 0.01) and the Control vs. Gi group (*p* < 0.01). No significant difference was observed between Control vs. Gq (*p* > 0.05) for the percentage of activated PV interneurons (Figure 4I, Gq > Control > Gi for PV activation).

These results demonstrate that chemogenetic modulation of insular PV interneurons selectively alters ethanol‐seeking behavior without affecting locomotion. Specifically, activation of PV+ interneurons (Gq‐DREADDs) reduces ethanol‐seeking responses. In contrast, their inhibition (Gi‐DREADDs) increases operant responding for ethanol, underscoring their essential role in regulating motivated and reward‐driven behaviors.

## 4 Discussion

Adolescence is a critical developmental window marked by heightened impulsivity and increased vulnerability to substance use, yet the neural mechanisms underlying these behaviors remain incompletely understood. In the present study, we investigated how the anterior insular cortex, with particular emphasis on parvalbumin (PV) GABAergic interneurons, contributes to impulsivity and ethanol‐related behaviors across development in mice [42]. Building on prior work demonstrating elevated impulsivity during adolescence [43, 44, 45], our findings provide mechanistic insight by linking developmental alterations in insular inhibitory microcircuits to behavioral vulnerability during this stage.

The omission‐contingency learning (OCL) task was used to assess inhibitory control and behavioral flexibility under a contingency shift. In this paradigm, animals were required to suppress a previously reinforced operant response and adapt their behavior to a reversed action–outcome contingency, in which reward delivery became contingent upon withholding responding for a predefined interval, while responses emitted during this interval reset the timer and delayed reinforcement. Thus, beyond measuring the ability to inhibit a prepotent response, the OCL task also probes sensitivity to changes in reinforcement contingencies, a critical component of adaptive behavioral control. Although the specific implementation used in the present study does not correspond to a single standardized protocol, it is conceptually grounded in well‐established omission‐based learning and response inhibition paradigms [46, 47, 48], as well as addiction‐related models of compulsive responding and impaired behavioral control [49, 50]. In addition, interval‐resetting responses were quantified as a direct measure of inappropriate responding and impaired inhibitory control.

The insular cortex has been consistently implicated in impulsivity, decision‐making, and alcohol‐related behaviors [45, 51, 52, 53]. Functional activation of this region enhances motivation for alcohol consumption and influences drinking severity [54], whereas chemogenetic manipulation of insular glutamatergic neurons modulates alcohol intake, impulsive behavior, and risk‐based decision‐making [36, 54, 55]. Our data extend this literature by identifying PV interneurons as a critical inhibitory component within the insular cortex that shapes impulsivity‐related behaviors and ethanol seeking. Developmental differences in insular c‐Fos activation patterns suggest that adolescence is characterized by an imbalance between excitation and inhibition, likely reflecting delayed maturation of inhibitory control mechanisms.

In the present study, c‐Fos expression was specifically assessed following the omission‐contingency learning (OCL) task to examine neuronal recruitment associated with inhibitory control. c‐Fos expression was not evaluated following quinine‐adulterated ethanol sessions, as the two‐bottle choice procedure involved repeated daily 4‐h drinking sessions followed by continued behavioral testing after quinine exposure, making tissue collection within the optimal temporal window for c‐Fos detection incompatible with the longitudinal behavioral design. Future studies specifically designed to capture neural activation during quinine‐adulterated ethanol intake will provide important insight into the circuit mechanisms underlying aversion‐resistant alcohol consumption.

PV interneurons play a central role in regulating cortical excitability and synchronizing pyramidal neuron output [31, 56]. Evidence from other cortical regions indicates that dysfunction of PV‐mediated inhibitory control contributes to impulsivity and compulsive behaviors [57]. Consistent with this framework, our findings support the idea that immature or underactive PV interneurons within the insular cortex during adolescence may promote heightened reward valuation, inefficient interoceptive processing, and impaired inhibitory control [33], [53, 58, 59]. Such inhibitory microcircuit dysfunctions may increase pyramidal neuron activity and contribute to cognitive and behavioral impairments associated with impulsivity [31, 56].

An important limitation of the present Fos analysis is the absence of a home‐cage control group, which precludes determining whether the age‐dependent differences observed in insular PV interneuron activation reflect pre‐existing developmental differences in baseline neuronal activity or differential recruitment induced by task performance. The present experimental design was specifically intended to capture experience‐dependent neuronal activation following the OCL procedure, allowing direct comparison between adolescent and adult mice under identical behavioral conditions. However, future studies incorporating untreated baseline controls will be important to dissociate intrinsic developmental differences in insular circuit activity from task‐evoked neuronal recruitment.

Chemogenetic manipulation further supports a causal role for insular PV interneurons in behavioral regulation. Altering PV interneuron activity in adult animals reproduced key features associated with adolescent‐like impulsivity, reinforcing the notion that proper maturation and engagement of these interneurons are essential for behavioral stability. These findings are consistent with evidence that late insular maturation and neuronal hyperexcitability contribute to vulnerability during adolescence [60, 61, 62].

An important limitation of the present study is that the open‐field assessment alone does not fully exclude the possibility that the reduced responding observed following Gq‐mediated activation of insular PV interneurons may partly reflect non‐specific suppression of operant or consummatory performance. However, several observations argue against a purely global motor impairment, including the absence of locomotor changes and the bidirectional behavioral effects observed following Gq and Gi manipulations of the same neuronal population. Consistent with this interpretation, previous studies have shown that chemogenetic activation of the anterior insula selectively reduces alcohol‐ and sucrose‐directed consumption while sparing locomotion and deprivation‐induced water intake, supporting a specific role of insular activity in the regulation of appetitive behavior [54]. Nevertheless, dedicated consummatory or operant control tasks will be important in future studies to more directly dissociate specific effects on reward‐directed behavior from broader performance‐related suppression. More broadly, this consideration aligns with current recommendations in the chemogenetic literature, which emphasize the importance of behavioral controls beyond locomotor assessment alone, particularly given potential off‐target effects associated with CNO metabolism [63, 64, 65].

The operant alcohol session used in the present study was intentionally designed as a probe of alcohol‐directed responding following a defined history of operant training and ethanol exposure, rather than as a conventional model of the acquisition or maintenance of alcohol self‐administration. This experimental strategy allows the assessment of alcohol‐seeking‐like responding under stable behavioral conditions while minimizing variability associated with acquisition processes. Importantly, prior behavioral history and ethanol pre‐exposure are well known to shape subsequent alcohol‐directed responding in operant paradigms [66, 67, 68], and operant responding does not necessarily correlate directly with ethanol consumption or preference measures [69]. Accordingly, the present findings should be interpreted as reflecting alcohol‐directed operant responding under a controlled behavioral history, rather than outcomes derived from a canonical operant self‐administration paradigm.

The chemogenetic experiments were designed as an initial proof‐of‐principle approach to test the causal contribution of insular PV interneurons to impulsivity‐related behavior. Although the bidirectional effects observed following excitation and inhibition of the same neuronal population provide internally consistent support for a circuit‐specific role, the relatively modest sample size warrants cautious interpretation of effect magnitude and generalizability. Future studies using larger cohorts and prospective power analyses will be important for further validating and refining the role of insular PV interneurons in inhibitory control and impulsivity‐related behaviors.

The forced ethanol exposure protocol was implemented to ensure controlled and consistent ethanol intake within the limited temporal window of adolescence. This approach minimizes variability associated with voluntary drinking paradigms and allows behavioral assessment within a defined developmental stage. In addition, this design is compatible with acute chemogenetic manipulation, avoiding potential confounds related to repeated DREADD activation across multiple sessions.

Interestingly, age‐related differences in insular activation were not observed following ethanol consumption. This may reflect alcohol‐induced increases in insular activity that obscure developmental differences, or methodological factors related to the duration of ethanol exposure. In parallel, reduced sensitivity to taste aversion in adolescents aligns with previous reports [26, 70, 71] and may be explained by ongoing maturation of insular circuits involved in aversive processing and taste–visceral integration [72, 73]. The insula undergoes dynamic, age‐dependent functional organization [70, 71, 74], supporting its role in aversive perception, conditioned taste aversion, and visceral signaling [73, 75, 76]. Experimental reductions in insular activity diminish aversive visceral responses [77, 78], further underscoring its relevance to ethanol‐related behaviors.

Overall, these findings highlight the insular cortex and its PV interneurons as key regulators of impulsivity and ethanol‐related behaviors across development. The maturation of inhibitory microcircuits, particularly PV‐mediated control of pyramidal neuron activity, appears central to the transition from adolescent vulnerability to adult behavioral stability. Together, these data identify insular PV interneurons as promising targets for interventions aimed at reducing impulsivity and mitigating vulnerability to alcohol misuse.

## 5 Conclusion

Our findings identify the insular cortex as a critical regulator of impulsivity and ethanol‐related behaviors, highlighting the essential contribution of insular parvalbumin‐positive (PV) interneurons to developmental vulnerability for alcohol use disorder (AUD). Given the insula's central role in integrating interoceptive, motivational, and aversive information, disruptions in cortical PV‐interneuron‐mediated inhibitory control may represent a key mechanism contributing to adolescent vulnerability to AUD. Future studies should examine the longitudinal developmental trajectories of insular GABAergic function, assess subregion‐specific circuit interactions, and explore how insular PV‐mediated inhibition shapes the maturation of reward and aversion pathways. Elucidating these mechanisms may enable the development of targeted strategies aimed at enhancing inhibitory control and reducing addiction risk during adolescence.

## Author Contributions

F. Bezerra: Conceptualization; Methodology; Investigation; Formal Analysis; Visualization; writing – original draft. G.J.D. Fernandes: Investigation; writing – review and editing. A. Anjos‐Santos: Investigation; writing – review and editing. C.A. Favoretto: Investigation; writing – review and editing. B.T. Rodolpho: writing – review and editing. P. Palombo: Visualization, writing – review and editing. V. Vialou: Resources; Supervision; writing – review and editing. K.P. Abrahao: Conceptualization; Funding Acquisition; Supervision; review and editing. F.C. Cruz: Conceptualization; Funding Acquisition; Project Administration; Supervision; writing – review and editing.

## Funding

This work was supported by Fundação de Amparo à Pesquisa do Estado de São Paulo, 2019/24073‐3, 2018/15505‐4, 2019/01686‐0. Conselho Nacional de Desenvolvimento Científico e Tecnológico, 314336/2023‐0. Coordenação de Aperfeiçoamento de Pessoal de Nível Superior, CAPES, Finance Code 001.

## Ethics Statement

All experimental procedures involving animals were conducted in accordance with the guidelines of the Principles of Care of Laboratory Animals. Protocols were approved by the Ethics Committee on Animal Use of the Federal University of São Paulo (CEUA #9191091219) and by the local Animal Experimentation Ethics Committee C2EA‐05 Charles Darwin of Sorbonne Université, under the European Council Directive 2010/63/EU, as authorized by the French Ministry of Higher Education and Research.

## Conflicts of Interest

The authors declare no conflicts of interest.

## Supporting information


**Figure S1:** Principal Component Analysis. Principal Component 3 (PC3) was loaded by consumption in the 3rd session of EtOH + Quinine (g/Kg/EtOH—SQ3) and by preference in this same session (%PREF—SQ3). Principal Component 4 (CP4) was formed by the consumption of ethanol in the 1st session of EtOH + Quinine (g/Kg/EtOH—SQ1). by preference in this same session (%PREF—SQ1) and by preference in the first ethanol session (%PREF—S1 Principal Component 5 (CP5)) was formed by the consumption in the 2nd session of EtOH (g/Kg/EtOH—S2), by the preference in this same session (%PREF—S2) and also by the consumption of ethanol in the 1st session of the Two‐Bottle Choice (g/Kg/EtOH—S1). *** *p* < 0.001. Adolescents, *n* = 8; Adults, *n* = 8. Data shown as means ± standard deviation of the mean (SEM).
**Figure S2:** Neuronal activation (number of Fos‐positive cells/mm2) during the last session of the two‐bottle free choice of ethanol intake (Adolescent mice in blue; Adult mice in red). *p > 0.05. Adolescents, *n* = 8; Adults, *n* = 8. Data shown as means ± standard deviation of the mean (SEM).
**Figure S3:** Representative images of Fos‐positive cells in the insular cortex. In A, illustration of a brain coronal slice, highlighting the insular cortex (in red) (adapted from Paxinos & Watson, 2007). In B, photomicrograph of the insular cortex of an adolescent mouse. In C, photomicrograph of the insular cortex of an adult mouse.
**Figure S4:** Locomotor activity in the open field. In A, total distance traveled (m) during the open field test. In B, average speed (m/s) of mice during the open field test. [control DREADD in black; inhibitory DREADD (Gi) in red; excitatory DREADD (Gq), in blue]. *p < 0.05; *n* = 4 mice per group. Data shown as means ± SEM.

**Data S1:** Supporting Information.

## Data Availability

The datasets generated and/or analyzed during the current study are available from the corresponding author upon reasonable request. All data supporting the findings of this study are included in the article and its Supporting Information.

## References

[1] WHO , Global alcohol action plan 2022‐2030 to strengthen implementation of the Global Strategy to Reduce the Harmful Use of Alcohol, 2021 accessed at, January 30, 2023, https://www.who.int/teams/mental‐health‐and‐substance‐use/alcohol‐drugs‐.

[2] WHO , “Global Status Report on Alcohol and Health 2018,” in Geneva: World Health Organization (World Health Organization, 2018), 65.

[3] UNODC , “World Drug Report 2018,” in World Drug Report (United Nations Office on Drugs and Crime, 2018), 34. available from, https://www.unodc.org/wdr2018.

[4] APA , Manual diagnóstico e estatístico de transtornos mentais: DSM‐5—5^a^ Edição (American Psychiatric Association, 2013).

[5] A. F. Carvalho , M. Heilig , A. Perez , C. Probst , and J. Rehm , “Alcohol Use Disorders,” Lancet 394, no. 10200 (2019): 781–792, 10.1016/S0140-6736(19)31775-1. 31478502

[6] J. P. Connor , P. S. Haber , and W. D. Hall , “Alcohol Use Disorders,” Lancet 387, no. 10022 (2016): 988–998, 10.1016/S0140-6736(15)00122-1. 26343838

[7] L. Kuhns , E. Kroon , H. Lesscher , G. Mies , and J. Cousijn , “Age‐Related Differences in the Effect of Chronic Alcohol on Cognition and the Brain: A Systematic Review,” Translational Psychiatry 12, no. 1 (2022): 345, 10.1038/s41398-022-02100-y. PMC9411553 36008381

[8] B. J. Casey , R. M. Jones , and T. A. Hare , “The Adolescent Brain,” Annals of the New York Academy of Sciences 1124 (2008): 111–126, 10.1196/ANNALS.1440.010. PMC2475802 18400927

[9] L. M. Squeglia , J. Jacobus , and S. F. Tapert , “The Effect of Alcohol Use on Human Adolescent Brain Structures and Systems,” in Handbook of Clinical Neurology, vol. 125 (2014), 501, 10.1016/B978-0-444-62,619-6.00028-8. PMC4321715 25307592

[10] A. Nath , S. G. Choudhari , S. U. Dakhode , A. Rannaware , and A. M. Gaidhane , “Substance Abuse Amongst Adolescents: An Issue of Public Health Significance,” Cureus 14, no. 11 (2022): e31193, 10.7759/cureus.31193. PMC9728192 36505140

[11] B. Lees , L. Mewton , L. A. Stapinski , L. M. Squeglia , C. D. Rae , and M. Teesson , “Neurobiological and Cognitive Profile of Young Binge Drinkers: A Systematic Review and Meta‐Analysis,” Neuropsychology Review 29, no. 3 (2019): 357–385, 10.1007/S11065-019-09411-W. PMC7231524 31512192

[12] A. M. Nawi , R. Ismail , F. Ibrahim , et al., “Risk and Protective Factors of Drug Abuse Among Adolescents: A Systematic Review,” BMC Public Health 21, no. 1 (2021): 2088, 10.1186/s12889-021-11906-2. PMC8590764 34774013

[13] I. N. Defoe , A. Khurana , L. M. Betancourt , H. Hurt , and D. Romer , “Cascades From Early Adolescent Impulsivity to Late Adolescent Antisocial Personality Disorder and Alcohol Use Disorder,” Journal of Adolescent Health 71, no. 5 (2022): 579–586, 10.1016/J.JADOHEALTH.2022.06.007. PMC11184504 35934585

[14] D. C. Fowles , “Psychophysiology and Psychopathology: A Motivational Approach,” Psychophysiology 25, no. 4 (1988): 373–391, 10.1111/J.1469-8986.1988.TB01873.X. 3051073

[15] I. Kovács , I. Demeter , Z. Janka , Z. Demetrovics , A. Maraz , and B. Andó , “Different Aspects of Impulsivity in Chronic Alcohol Use Disorder With and Without Comorbid Problem Gambling,” PLoS One 15, no. 1 (2020): e0227645, 10.1371/JOURNAL.PONE.0227645. PMC6992191 31999707

[16] C. L. Vaughan , B. L. Stangl , M. L. Schwandt , K. M. Corey , C. S. Hendershot , and V. A. Ramchandani , “The Relationship Between Impaired Control, Impulsivity and Alcohol Self‐Administration in Non‐Dependent Drinkers,” Experimental and Clinical Psychopharmacology 27, no. 3 (2019): 236–246, 10.1037/PHA0000247. PMC6776085 30688502

[17] Y. Zhang , X. Zhong , Y. Shao , and J. Gong , “Insula Connectivity Abnormalities Predict Impulsivity in Chronic Heroin Use Disorder: A Cross‐Sectional Resting‐State fMRI Study,” Brain Sciences 13, no. 11 (2023): 1508, 10.3390/BRAINSCI13111508. PMC10669645 38002468

[18] M. V. Elliott , S. A. S. Esmail , K. S. Weiner , and S. L. Johnson , “Neuroanatomical Correlates of Emotion‐Related Impulsivity,” Biological Psychiatry 93, no. 6 (2023): 566–574, 10.1016/J.BIOPSYCH.2022.07.018. PMC9898470 36244800

[19] A. Belin‐Rauscent , M. L. Daniel , M. Puaud , et al., “Impulsivity Is Predicted by the Thinness of the Insular Cortex in Rats,” Molecular Psychiatry 21, no. 4 (2016): 445, 10.1038/MP.2016.32. 26996229

[20] A. Belin‐Rauscent , M. L. Daniel , M. Puaud , et al., “From Impulses to Maladaptive Actions: The Insula Is a Neurobiological Gate for the Development of Compulsive Behavior,” Molecular Psychiatry 21, no. 4 (2016): 491–499, 10.1038/MP.2015.140. 26370145

[21] T. Pattij , D. Schetters , and A. N. M. Schoffelmeer , “Dopaminergic Modulation of Impulsive Decision Making in the Rat Insular Cortex,” Behavioural Brain Research 270 (2014): 118–124, 10.1016/J.BBR.2014.05.010. 24837747

[22] C. C. Probst and T. Van Eimeren , “The Functional Anatomy of Impulse Control Disorders,” Current Neurology and Neuroscience Reports 13, no. 10 (2013): 386, 10.1007/S11910-013-0386-8. PMC3779310 23963609

[23] M. Tanzer , M. Derome , L. Morosan , G. Salaminios , and M. Debbané , “Cortical Thickness of the Insula and Prefrontal Cortex Relates to Externalizing Behavior: Cross‐Sectional and Prospective Findings,” Development and Psychopathology 33, no. 4 (2021): 1437–1447, 10.1017/S0954579420000619. 32638690

[24] K. S. Jadhav , A. P. Bernheim , L. Aeschlimann , et al., “Reversing Anterior Insular Cortex Neuronal Hypoexcitability Attenuates Compulsive Behavior in Adolescent Rats,” Proceedings. National Academy of Sciences. United States of America 119, no. 21 (2022): e2121247119, 10.1073/pnas.2121247119. PMC9173752 35584117

[25] C. Tai , Y. Abe , R. E. Westenbroek , T. Scheuer , and W. A. Catterall , “Impaired Excitability of Somatostatin‐ and Parvalbumin‐Expressing Cortical Interneurons in a Mouse Model of Dravet Syndrome,” Proceedings of the National Academy of Sciences of the United States of America 111, no. 30 (2014): E3139–E3148, 10.1073/PNAS.1411131111. PMC4121787 25024183

[26] S. W. Centanni , A. C. Janes , D. L. Haggerty , B. Atwood , and F. W. Hopf , “Better Living Through Understanding the Insula: Why Subregions Can Make All the Difference,” Neuropharmacology 198 (2021): 108765, 10.1016/J.NEUROPHARM.2021.108765. PMC13284909 34461066

[27] E. J. Campbell , J. P. M. Flanagan , L. C. Walker , M. K. R. I. Hill , N. J. Marchant , and A. J. Lawrence , “Anterior Insular Cortex Is Critical for the Propensity to Relapse Following Punishment‐Imposed Abstinence of Alcohol Seeking,” Journal of Neuroscience 39, no. 6 (2019): 1077–1087, 10.1523/JNEUROSCI.1596-18.2018. PMC6363928 30509960

[28] H. Chen , D. He , and A. W. Lasek , “Repeated Binge Drinking Increases Perineuronal Nets in the Insular Cortex,” Alcoholism, Clinical and Experimental Research 39, no. 10 (2015): 1930–1938, 10.1111/ACER.12847. PMC4592458 26332441

[29] H. Chen and A. W. Lasek , “Perineuronal Nets in the Insula Regulate Aversion‐Resistant Alcohol Drinking,” Addiction Biology 25, no. 6 (2020): e12821, 10.1111/ADB.12821. PMC7032993 31433552

[30] J. M. Blasco‐Ibáñez and T. F. Freund , “Synaptic Input of Horizontal Interneurons in Stratum Oriens of the Hippocampal CA1 Subfield: Structural Basis of Feed‐Back Activation,” European Journal of Neuroscience 7, no. 10 (1995): 2170–2180, 10.1111/J.1460-9568.1995.TB00638.X. 8542073

[31] R. S. Duman , G. Sanacora , and J. H. Krystal , “Altered Connectivity in Depression: GABA and Glutamate Neurotransmitter Deficits and Reversal by Novel Treatments,” Neuron 102, no. 1 (2019): 75–90, 10.1016/J.NEURON.2019.03.013. PMC6450409 30946828

[32] J. M. Mcnally and R. W. Mccarley , “Gamma Band Oscillations: A Key to Understanding Schizophrenia Symptoms and Neural Circuit Abnormalities,” Current Opinion in Psychiatry 29, no. 3 (2017): 202–210, 10.1097/YCO.0000000000000244.Gamma. PMC4901383 26900672

[33] B. R. Ferguson and W. J. Gao , “PV Interneurons: Critical Regulators of E/I Balance for Prefrontal Cortex‐Dependent Behavior and Psychiatric Disorders,” Frontiers in Neural Circuits 12 (2018): 37, 10.3389/FNCIR.2018.00037. PMC5964203 29867371

[34] J. Rice , L. Coutellier , J. L. Weiner , and C. Gu , “Region‐Specific Interneuron Demyelination and Heightened Anxiety‐Like Behavior Induced by Adolescent Binge Alcohol Treatment,” Acta Neuropathologica Communications 7, no. 1 (2019): 173, 10.1186/S40478-019-0829-9. PMC6842209 31703603

[35] A. A. Jaramillo , V. E. Agan , V. H. Makhijani , S. Pedroza , Z. A. McElligott , and J. Besheer , “Functional Role for Suppression of the Insular–Striatal Circuit in Modulating Interoceptive Effects of Alcohol,” Addiction Biology 23, no. 5 (2018): 1020–1031, 10.1111/adb.12551. PMC5871527 28960802

[36] A. A. Jaramillo , K. Van Voorhies , P. A. Randall , and J. Besheer , “Silencing the Insular‐Striatal Circuit Decreases Alcohol Self‐Administration and Increases Sensitivity to Alcohol,” Behavioural Brain Research 348 (2018): 74–81, 10.1016/J.BBR.2018.04.007. PMC5993624 29660441

[37] A. A. Jaramillo , P. A. Randall , S. Stewart , B. Fortino , K. Van Voorhies , and J. Besheer , “Functional Role for Cortical‐Striatal Circuitry in Modulating Alcohol Self‐Administration,” Neuropharmacology 130 (2018): 42–53, 10.1016/j.neuropharm.2017.11.035. PMC5891728 29183687

[38] C. Tanahira , S. Higo , K. Watanabe , et al., “Parvalbumin Neurons in the Forebrain as Revealed by Parvalbumin‐Cre Transgenic Mice,” Neuroscience Research 63, no. 3 (2009): 213–223, 10.1016/J.NEURES.2008.12.007. 19167436

[39] L. Spear , “Modeling Adolescent Development and Alcohol Use in Animals,” Alcohol Research & Health 24, no. 2 (2000): 115–123. PMC6713014 11199278

[40] J. A. Jablonka , R. Binkowski , M. Kazmierczak , et al., “The Role of Interhemispheric Interactions in Cortical Plasticity,” Frontiers in Neuroscience 15 (2021): 631328, 10.3389/fnins.2021.631328. PMC8299724 34305511

[41] A. Mundorf and S. Ocklenburg , “Hemispheric Asymmetries in Mental Disorders: Evidence From Rodent Studies,” Journal of Neural Transmission 130, no. 9 (2023): 1153–1165, 10.1007/S00702-023-02610-Z. PMC10460727 36842091

[42] S. A. Jones , J. M. Lueras , and B. J. Nagel , “Effects of Binge Drinking on the Developing Brain: Studies in Humans,” Alcohol Research: Current Reviews 39, no. 1 (2018): 87–96. 10.35946/arcr.v39.1.11 PMC6104956 30557151

[43] B. Lees , L. R. Meredith , A. E. Kirkland , B. E. Bryant , and L. M. Squeglia , “Effect of Alcohol Use on the Adolescent Brain and Behavior,” Pharmacology, Biochemistry, and Behavior 192 (2020): 172906, 10.1016/J.PBB.2020.172906. PMC7183385 32179028

[44] J. Evenden , “Impulsivity: A Discussion of Clinical and Experimental Findings,” Journal of Psychopharmacology 13, no. 2 (1999): 180–192, 10.1177/026988119901300211. 10475725

[45] J. W. Dalley and T. W. Robbins , “Fractionating Impulsivity: Neuropsychiatric Implications,” Nature Reviews. Neuroscience 18, no. 3 (2017): 158–171, 10.1038/NRN.2017.8. 28209979

[46] D. M. Eagle and T. W. Robbins , “Inhibitory Control in Rats Performing a Stop‐Signal Reaction‐Time Task: Effects of Lesions of the Medial Striatum and d‐Amphetamine,” Behavioral Neuroscience 117, no. 6 (2003): 1302–1317, 10.1037/0735-7044.117.6.1302. 14674849

[47] A. Bari , T. W. Robbins , and J. W. Dalley , “Impulsivity,” NeuroMethods 53 (2011): 379–401, 10.1007/978-1-60,761-934-5_14.

[48] E. Coutureau , F. Esclassan , G. Di Scala , and A. R. Marchand , “The Role of the Rat Medial Prefrontal Cortex in Adapting to Changes in Instrumental Contingency,” PLoS One 7, no. 4 (2012): e33302, 10.1371/JOURNAL.PONE.0033302. PMC3319541 22496747

[49] C. L. Pickens , P. Kallenberger , A. Pajser , and H. Fisher , “Voluntary Alcohol Access During Adolescence/Early Adulthood, but Not During Adulthood, Causes Faster Omission Contingency Learning,” Behavioural Brain Research 370 (2019): 111918, 10.1016/j.bbr.2019.111918. PMC6590999 31095991

[50] C. L. Pickens , A. Cook , and B. Gaeddert , “Dose‐Dependent Effects of Alcohol Injections on Omission‐Contingency Learning Have an Inverted‐U Pattern,” Behavioural Brain Research 392 (2020): 112736, 10.1016/J.BBR.2020.112736. PMC7727456 32497681

[51] F. Dambacher , A. T. Sack , J. Lobbestael , A. Arntz , S. Brugman , and T. Schuhmann , “Out of Control: Evidence for Anterior Insula Involvement in Motor Impulsivity and Reactive Aggression,” Social Cognitive and Affective Neuroscience 10, no. 4 (2015): 508–516, 10.1093/SCAN/NSU077. PMC4381232 24837479

[52] V. Menon , G. Gallardo , M. A. Pinsk , et al., “Microstructural Organization of Human Insula is Linked to Its Macrofunctional Circuitry and Predicts Cognitive Control,” eLife 9 (2020): e53470, 10.7554/eLife.53470. PMC7308087 32496190

[53] I. Molnar‐Szakacs and L. Q. Uddin , “Anterior Insula as a Gatekeeper of Executive Control,” Neuroscience and Biobehavioral Reviews 139 (2022): 104736, 10.1016/J.NEUBIOREV.2022.104736. 35700753

[54] M. Haaranen , G. Scuppa , S. Tambalo , et al., “Anterior Insula Stimulation Suppresses Appetitive Behavior While Inducing Forebrain Activation in Alcohol‐Preferring Rats,” Translational Psychiatry 10, no. 1 (2020): 150, 10.1038/S41398-020-0833-7. PMC7235223 32424183

[55] H. Mizoguchi , K. Katahira , A. Inutsuka , et al., “Insular Neural System Controls Decision‐Making in Healthy and Methamphetamine‐Treated Rats,” Proceedings of the National Academy of Sciences of the United States of America 112, no. 29 (2015): E3930–E3939, 10.1073/pnas.1418014112. PMC4517258 26150496

[56] F. Donato , S. B. Rompani , and P. Caroni , “Parvalbumin‐Expressing Basket‐Cell Network Plasticity Induced by Experience Regulates Adult Learning,” Nature 2013 504:7479 504, no. 7479 (2013): 272–276, 10.1038/nature12866. 24336286

[57] H. Kim , S. Ährlund‐Richter , X. Wang , K. Deisseroth , and M. Carlén , “Prefrontal Parvalbumin Neurons in Control of Attention,” Cell 164, no. 1–2 (2016): 208–218, 10.1016/J.CELL.2015.11.038. PMC4715187 26771492

[58] A. Yiannakas , S. Kolatt Chandran , H. Kayyal , N. Gould , M. Khamaisy , and K. Rosenblum , “Parvalbumin Interneuron Inhibition Onto Anterior Insula Neurons Projecting to the Basolateral Amygdala Drives Aversive Taste Memory Retrieval,” Current Biology 31, no. 13 (2021): 2770–2784.e6, 10.1016/j.cub.2021.04.010. 33930301

[59] H. Kayyal , A. Yiannakas , S. K. Chandran , M. Khamaisy , V. Sharma , and K. Rosenblum , “Activity of Insula to Basolateral Amygdala Projecting Neurons Is Necessary and Sufficient for Taste Valence Representation,” Journal of Neuroscience 39, no. 47 (2019): 9369–9382, 10.1523/JNEUROSCI.0752-19.2019. PMC6867822 31597726

[60] Y. X. Luo , E. Galaj , and Y. Y. Ma , “Differential Alterations of Insular Cortex Excitability After Adolescent or Adult Chronic Intermittent Ethanol Administration in Male Rats,” Journal of Neuroscience Research 99, no. 2 (2020): 649–661, 10.1002/JNR.24737. PMC8443162 33094531

[61] C. A. Dannenhoffer , A. Gómez‐A , V. A. Macht , et al., “Impact of Adolescent Intermittent Ethanol Exposure on Interneurons and Their Surrounding Perineuronal Nets in Adulthood,” Alcoholism, Clinical and Experimental Research 46, no. 5 (2022): 759–769, 10.1111/ACER.14810. PMC9117471 35307830

[62] F. T. Crews , D. L. Robinson , L. J. Chandler , et al., “Mechanisms of Persistent Neurobiological Changes Following Adolescent Alcohol Exposure: NADIA Consortium Findings,” Alcoholism, Clinical and Experimental Research 43, no. 9 (2019): 1806–1822, 10.1111/ACER.14154. PMC6758927 31335972

[63] J. L. Gomez , J. Bonaventura , W. Lesniak , et al., “Chemogenetics Revealed: DREADD Occupancy and Activation via Converted Clozapine,” Science 357, no. 6350 (2017): 503–507, 10.1126/SCIENCE.AAN2475. PMC7309169 28774929

[64] M. Jendryka , M. Palchaudhuri , D. Ursu , et al., “Pharmacokinetic and Pharmacodynamic Actions of Clozapine‐N‐Oxide, Clozapine, and Compound 21 in DREADD‐Based Chemogenetics in Mice,” Scientific Reports 9 (2019): 4522, 10.1038/s41598-019-41088-2. PMC6418145 30872749

[65] S. V. Mahler and G. Aston‐Jones , “CNO Evil? Considerations for the Use of DREADDs in Behavioral Neuroscience,” Neuropsychopharmacology 43, no. 5 (2018): 934–936, 10.1038/npp.2017.299. PMC5854815 29303143

[66] P. H. Janak and N. Chaudhri , “The Potent Effect of Environmental Context on Relapse to Alcohol‐Seeking After Extinction,” Open Addiction Journal 3, no. 1 (2010): 76–87, 10.2174/1874941001003010076. PMC2995923 21132088

[67] H. H. Samson , “Initiation of Ethanol Reinforcement Using a Sucrose‐Substitution Procedure in Food‐ and Water‐Sated Rats,” Alcoholism, Clinical and Experimental Research 10, no. 4 (1986): 436–442, 10.1111/J.1530-0277.1986.TB05120.X. 3530023

[68] H. L. June , K. L. Foster , P. F. McKay , et al., “The Reinforcing Properties of Alcohol Are Mediated by GABA(A1) Receptors in the Ventral Pallidum,” Neuropsychopharmacology 28, no. 12 (2003): 2124–2137, 10.1038/SJ.NPP.1300239. 12968126

[69] C. S. R. Li , X. Luo , P. Yan , K. Bergquist , and R. Sinha , “Altered Impulse Control in Alcohol Dependence: Neural Measures of Stop Signal Performance,” Alcoholism, Clinical and Experimental Research 33, no. 4 (2009): 740–750, 10.1111/J.1530-0277.2008.00891.X. PMC2697053 19170662

[70] J. Saalfield and L. Spear , “The Ontogeny of Ethanol Aversion,” Physiology & Behavior 156 (2016): 164–170, 10.1016/J.PHYSBEH.2016.01.011. PMC4753083 26774181

[71] J. K. Gore‐Langton , E. I. Varlinskaya , D. F. Werner , and N. of A. D. in A. C. (NADIA) , “Ethanol‐Induced Conditioned Taste Aversion and Associated Neural Activation in Male Rats: Impact of Age and Adolescent Intermittent Ethanol Exposure,” PLoS One 17, no. 12 (2022): e0279507, 10.1371/JOURNAL.PONE.0279507. PMC9778589 36548243

[72] H. Ogawa , K. Hasegawa , S. Ofcfctawa , and I. Ikeda , “GABAergic Inhibition and Modifications of Taste Responses in the Cortical Taste Area in Rats,” Neuroscience Research 32, no. 1 (1998): 85–95, 10.1016/S0168-0102(98)00071-6. 9831255

[73] A. H. Jung , C. T. King , G. D. Blonde , et al., “A Subregion of Insular Cortex Is Required for Rapid Taste‐Visceral Integration and Consequent Conditioned Taste Aversion and Avoidance Expression in Rats,” eNeuro 9, no. 4 (2022): 527–2022, 10.1523/ENEURO.0527-21.2022. PMC9267001 35641228

[74] E. L. Dennis , N. Jahanshad , K. L. McMahon , et al., “Development of Brain Structural Connectivity Between Ages 12 and 30: A 4‐Tesla Diffusion Imaging Study in 439 Adolescents and Adults,” NeuroImage 64, no. 1 (2013): 671–684, 10.1016/J.NEUROIMAGE.2012.09.004. PMC3603574 22982357

[75] M. Contreras , F. Ceric , and F. Torrealba , “Inactivation of the Interoceptive Insula Disrupts Drug Craving and Malaise Induced by Lithium,” Science (1979) 318, no. 5850 (2007): 655–658, 10.1126/SCIENCE.1145590. 17962567

[76] F. Bermudez‐Rattoni , “The Forgotten Insular Cortex: Its Role on Recognition Memory Formation,” Neurobiology of Learning and Memory 109 (2014): 207–216, 10.1016/J.NLM.2014.01.001. 24406466

[77] B. T. Stone , J. Y. Lin , A. Mahmood , A. J. Sanford , and D. B. Katz , “LiCl‐Induced Sickness Modulates Rat Gustatory Cortical Responses,” PLoS Biology 20, no. 7 (2022): e3001537, 10.1371/JOURNAL.PBIO.3001537. PMC9352195 35877759

[78] A. D. Craig , “How Do You Feel? Interoception: The Sense of the Physiological Condition of the Body,” Nature Reviews. Neuroscience 3, no. 8 (2002): 655–666, 10.1038/NRN894. 12154366

