# Supplementary material for: The Role of Anterior Insular Parvalbumin GABAergic Interneurons in Impulsivity‐Related and Alcohol‐Seeking Behaviors
Source: CNS Neurosci Ther. 2026 Sep 28;32(10):e71138. doi: 10.1002/cns.71138 (PMC13620219; doi:10.1002/cns.71138)
Supplement: Supplementary file 1 [file CNS-32-e71138-s002.docx]

**SUPPLEMENTARY MATERIAL**

1. **Supplementary Description of General Experimental Procedures**

**1.1	Omission-Contingency Learning (OCL) Test**

The OCL test was performed in operant conditioning chambers (170 × 150 × 200 mm, Med Associates®, VT, USA) placed inside sound-attenuated boxes. Each chamber was equipped with a yellow cue light (7.5 W) positioned above the active nose-poke (ANP) aperture, a white house light (7.5 W; 12 lux), and a single ANP positioned 45 mm above the floor on the same wall as the reward magazine. The magazine was connected via polyethylene tubing to a pellet dispenser (ENV-203IR, Med Associates®). Chambers and dispensers were controlled by MED-PC IV software, which recorded operant responses and delivered both cue light signals (3 s) and sucrose pellets (20 mg; Bio-Serv, Frenchtown, NJ, USA).

Animals were trained under schedules of increasing fixed-ratio (FR) reinforcement, consisting of three consecutive days of FR1, followed by three days of FR3, and three days of FR5. The test day consisted of a single 30-minute session conducted 24 hours after the final FR5 session. During the test, the software was programmed to deliver one sucrose pellet every 30 s; however, any ANP response performed before pellet delivery reset the 30‑s interval. The primary outcomes measured were the total number of ANP responses and the number of rewards (REW) earned.

The omission-contingency learning (OCL) paradigm was developed in our laboratory to assess inhibitory control and behavioral flexibility under changing action–outcome contingencies.

**1.2	Two-bottle choice and quinine adulterated drinking sessions**

To evaluate alcohol consumption and preference, mice were individually housed in cages equipped with two 50‑ml conical plastic bottles fitted with stainless-steel sipper tubes and two ball-valve nipples (Bonther, Ribeirão Preto, Brazil). Animals had simultaneous access to one bottle containing tap water and another containing a 10% (v/v) ethanol solution. Twelve daily drinking sessions were conducted, each lasting 4 h (08:30–12:30). During the first six sessions (S1–S6), mice were presented with water and 10% ethanol. Subsequently, animals underwent three quinine-adulteration sessions in which the ethanol bottle contained 10% ethanol mixed with increasing concentrations of quinine (0.6, 0.8, and 1.0 mM across consecutive sessions). After the quinine phase, mice completed three additional sessions with access to water and 10% ethanol. The variables measured were alcohol consumption (g / kg) and ethanol preference, calculated as: [(volume of ethanol consumed) / (volume of alcohol solution + volume of water consumed)] × 100.

**1.3	Immunofluorescence**

Coronal brain sections (30 µm) were collected on a cryostat and processed for immunofluorescence as previously described by Anjos-Santos et al. (2026), with modifications. Sections were rinsed five times (5 min each) in 0.1 M phosphate buffer (PB). Antigen retrieval was performed by incubating the sections in 0.01 M sodium citrate buffer (pH 8.0) at 70 °C for 20 min, followed by a 10-minute cooldown at room temperature. Sections were then washed in PBS and incubated in 1% hydrogen peroxide for 20 min under gentle agitation. After additional washes, sections were incubated for 2 h with biotinylated goat anti-mouse IgG (1:1000; **catalog #BA-9200; Vector Laboratories, Burlingame, CA, USA**) prepared in PB containing 1% Tween-20 and 3% normal goat serum. Following PB washes (5 × 5 min), sections were incubated for 48 h at 4 °C with combinations of the following primary antibodies diluted in PB containing 1% Tween-20 and 2% powdered milk: anti-Fos (1:3000; rabbit, **catalog #2250; Cell Signaling Technology, Danvers, MA, USA,** or mouse, **catalog #ab208942; Abcam, Cambridge, UK**), anti-NeuN (1:2000; mouse, **catalog #MAB377; Millipore, Temecula, CA, USA**), and anti-parvalbumin (1:3500; rabbit, **catalog #ab11427; Abcam, Cambridge, UK)**.

After primary antibody incubation, sections were washed and incubated for 2 h at room temperature with Alexa Fluor 488–conjugated secondary antibodies (1:200; goat anti-rabbit IgG, **catalog #A-11008**, or goat anti-mouse IgG, **catalog #A-11001**; Invitrogen, Carlsbad, CA, USA) or Alexa Fluor 594–conjugated secondary antibodies (1:1000; goat anti-mouse IgG, **catalog #A-11005**, or goat anti-rabbit IgG, **catalog #A-11012**; Invitrogen, Carlsbad, CA, USA), protected from light. Sections were then rinsed and incubated with DAPI (1:10,000; **catalog #D1306; Invitrogen, Carlsbad, CA, USA**). After additional PB washes, sections were mounted on gelatin-coated slides, air-dried, and coverslipped with Fluoromount mounting medium (**catalog #F4680; Sigma-Aldrich, St. Louis, MO, USA**). Slides were stored horizontally at 4–8 °C for 48 h before being sealed with nail polish.

Images were acquired using a Zeiss Axio Imager D2™ fluorescence microscope equipped with an Axiocam 503 color camera and controlled by Zen Blue (Carl Zeiss) software. The microscope light intensity was kept constant across all acquisitions, while the exposure time was automatically adjusted for each field of interest and each fluorescence channel. Objectives used for imaging included an E.C. Pan Neofluar 20x/0,5 (Zeiss). Percent neuronal activation was quantified using the formulas: [(overlap of cells positive for Fos and specific neuronal marker (NeuN) / number of cells positive for NeuN) * 100]. The calculation of the percentage of PV-activated cells was performed using the following formula: [(overlap of cells positive for Fos and PV+) / number of cells PV+) * 100].

**1.4	Experimental Design**

In Experiment 1, adolescent mice (from postnatal day (PND) 28) and adult mice (from PND 64) underwent omission-contingency learning protocol. In summary, the animals were submitted to 2 habituation sessions using a food magazine procedure. Next, mice were trained to receive a sucrose pellet during nine sessions under a fixed ratio (FR) schedule. Subsequently, the animals were tested in a single session to evaluate impulsive-related behavior. After 48h intervals, mice underwent a two-bottle choice protocol. Subjects were exposed to 2 sessions of habituation with water and 12 sessions of intermittent access. After the last session, mice were euthanized, and the brain was collected for Fos expression in the insular cortex via immunohistochemistry analysis. Created with BioRender.com.

In Experiment 2, adolescent mice (from postnatal day (PND) 28) and adult mice (from PND 64) underwent omission-contingency learning protocol. In summary, the animals were submitted to 2 habituation sessions using a food magazine procedure. Next, mice were trained to receive a sucrose pellet during nine sessions under a fixed ratio (FR) schedule. Finally, the animals were tested in a single session to evaluate impulsive-related behavior. After the test session, mice were euthanized, and the brain was collected for Fos and Parvalbumin expression in the insular cortex through immunofluorescence analysis. Created with BioRender.com.

In Experiment 3, transgenic mice underwent stereotaxic surgery to inject a viral vector for DREADDS expression in the insular cortex on postnatal day (PND) 35. After 15 days of recovery, animals were submitted to an omission-contingency learning protocol. In summary, mice underwent two habituation sessions using a food magazine procedure. Next, subjects were trained to receive a sucrose pellet during nine sessions under a fixed ratio (FR) schedule. Subsequently, the animals received an intraperitoneal injection of CNO (1 mg/kg) and, thirty minutes later, were tested in a single session to evaluate impulsive-related behavior. Created with BioRender.com

In Experiment 4, transgenic mice underwent stereotaxic surgery to inject a viral vector for DREADDS expression in the insular cortex on postnatal day (PND) 4. After 21 days of recovery, animals were submitted to an omission-contingency learning protocol. In summary, mice underwent two habituation sessions using a food magazine procedure. Next, subjects were trained to receive a sucrose pellet during nine sessions under a fixed ratio (FR) schedule. Subsequently, the animals received an intraperitoneal injection of CNO (1 mg/kg) and, thirty minutes later, were tested in a single session to evaluate impulsive-related behavior. The next day, mice underwent a self-administration protocol with four sessions of solution habituation on their home cages and one test session under an FR schedule. Subjects received an intraperitoneal injection of CNO (1 mg/kg) thirty minutes before the test session. After the last session, mice were euthanized, and the brain was collected for Fos expression in the insular cortex through immunofluorescence analysis. Created with BioRender.com.

**1.5	Immunohistochemistry**

Coronal brain slices (30 µm) containing the insular cortex (+1.54 mm to +1.94 mm relative to bregma) were collected stereotaxic coordinates according to the Paxinos and Franklin (2001) atlas, and were processed for Fos immunohistochemistry as previously described by Palombo *et al*. (2024). Sections containing the regions of interest were rinsed in 0.1 M PBS, incubated in 3% hydrogen peroxide for 20 min, and washed again. Slices were then incubated for 24 hours at 4°C in rabbit anti-Fos primary antibody (1:4000; Cell Signaling Technology) diluted in blocking solution (0.25% Triton X-100 and 3% normal goat serum in PBS). After washing, sections were incubated for 2 hours at room temperature with biotinylated anti-rabbit secondary antibody (1:1000; Vector Laboratories). Following additional washes, slices were incubated for 90 min in an avidin‑biotin peroxidase solution (ABC Elite Kit, Vector Laboratories). Fos immunoreactivity was visualized using 3,3′‑diaminobenzidine (DAB) for ~10 min. Sections were then washed, mounted on gelatin/chromic aluminum‑subbed slides, dried, and dehydrated through ascending ethanol concentrations and xylol. Slides were coverslipped with Entellan (Merck).

In an E.C. Pan Neofluar 20x/0,5 (Zeiss) objective. Fos‑positive nuclei were quantified from eight hemispheres (four slices per mouse) using QuPath software [2]. Image acquisition and quantification were performed in a blinded, independent manner of the experimental group.

**1.6**	**Surgical procedures**

Stereotaxic surgeries were performed on PV‑Cre mice (PND 35) under isoflurane anesthesia (4% induction, 1.5–3% maintenance), with subcutaneous lidocaine administered for local analgesia. Cre-dependent adeno-associated viral (AAV) vectors were obtained from Addgene (Watertown, MA, USA). Chemogenetic inhibition and excitation were achieved using AAV1-hSyn-DIO-hM4D(Gi)-mCherry (Addgene viral prep #44362-AAV1; RRID: Addgene_44362) and AAV1-hSyn-DIO-hM3D(Gq)-mCherry (Addgene viral prep #44361-AAV1; RRID: Addgene_44361), respectively. AAV1-EF1a-DIO-eYFP (Addgene viral prep #27056-AAV1; RRID: Addgene_27056) was used as a Cre-dependent fluorescent control vector. All viruses were unilaterally injected (0.2 µl/hemisphere) into the anterior insular cortex (coordinates relative to bregma: AP +1.7 mm, ML ±2.1 mm, DV −3.3 mm). Postoperative care included intramuscular streptomycin/penicillin (560 mg/ml/kg) (Poly Antibiotic, Fort Dodge) and subcutaneous flunixin meglumine (0.5 mg/ml/kg) (Schering-Plough). Viral constructs driven by synapsin or EF1α promoters ensured neuron‑specific expression, enabling chemogenetic manipulation via DREADDs [4]. Histological verification confirmed viral spread and targeting accuracy. Injections were counterbalanced between left and right hemispheres to account for functional lateralization of the insular cortex.

2. **Supplementary Results**
   2.1. **Experiment 2**

*Neuronal activation:* Unpaired Student T-test did not detect a significant difference between adolescent and adult experimental groups for the expression of Fos-positive cells [*T*(14) = 0.60; *p* = 0.556], as observed in **figure S2**. Representative images of mouse brain coronal slices containing the insular cortex immunostained for the Fos protein are shown in **figure S3**.

2.2. **Experiment 4**

*Evaluation of inhibitory (Gi) and excitatory (Gq) DREADD expression effects in the locomotor activity of mice:* ANOVA analysis did not reveal any significant differences between groups for the total distance moved [F(2,9) = 0.6351; *p* = 0.5520] or average speed in the open field [F(2,20) = 0.2183; *p* = 0.8080], as shown in **figure S4**.
