# Supplementary material for: The Role of Anterior Insular Parvalbumin GABAergic Interneurons in Impulsivity‐Related and Alcohol‐Seeking Behaviors
Source: CNS Neurosci Ther. 2026 Sep 28;32(10):e71138. doi: 10.1002/cns.71138 (PMC13620219; doi:10.1002/cns.71138)

Supplementary Results

Experiment 1: Figure S1

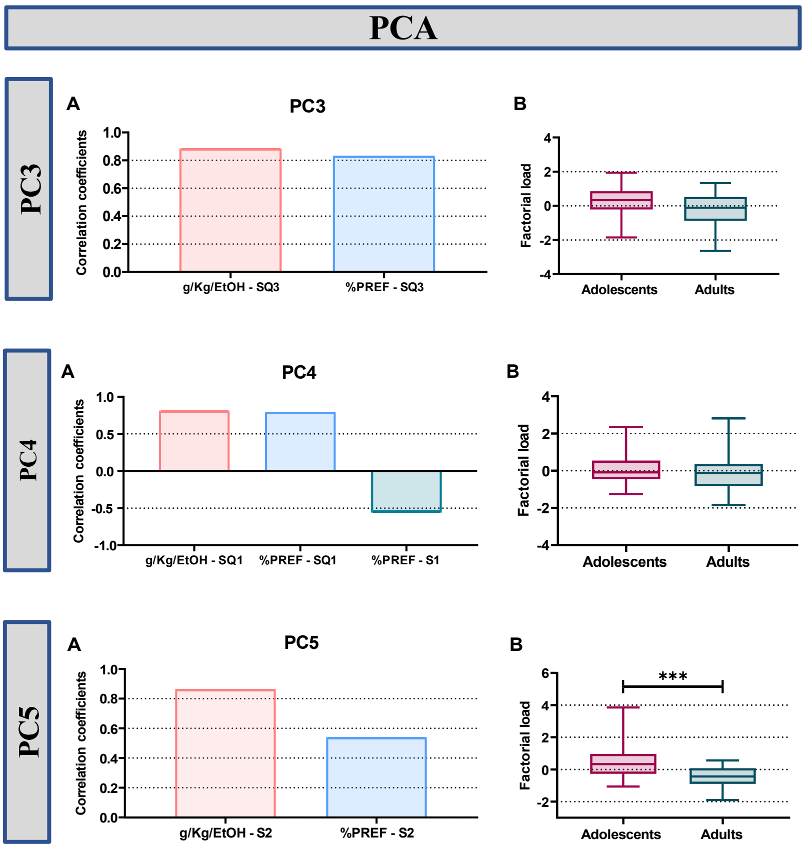

Experiment 2: Figure S2

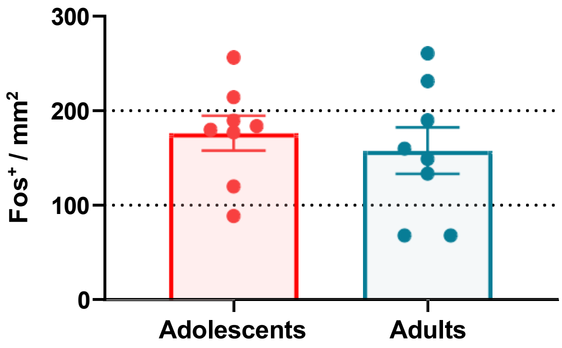

Experiment 2: Figure S3

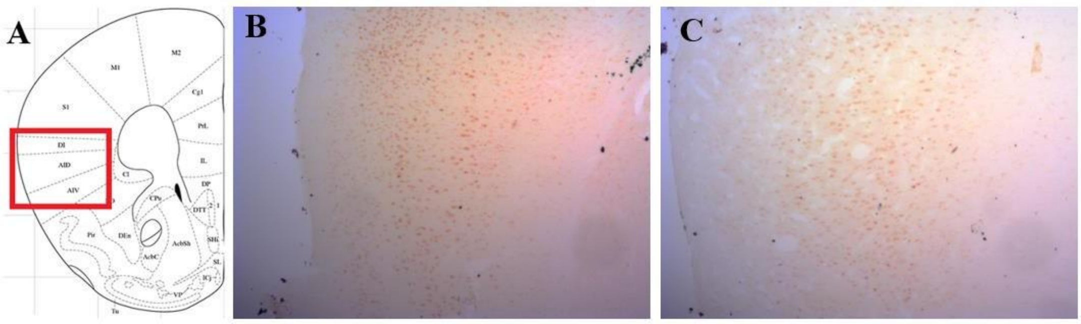

Experiment 4: S4

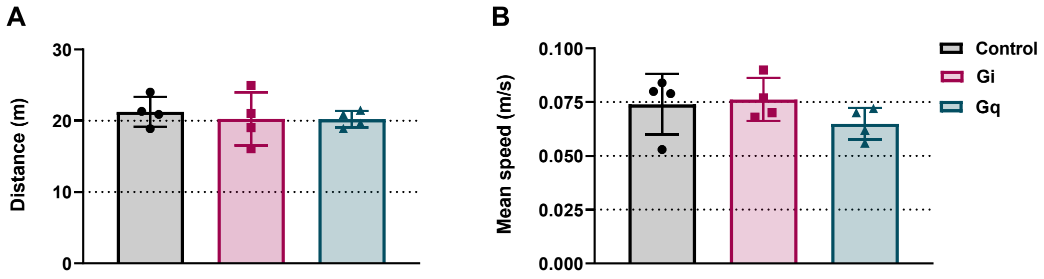

Supplement: Supplementary file 2 [file CNS-32-e71138-s001.zip › cns71138-sup-0002-Supinfo1.pdf]
